# Incidence of Microalbuminuria and Factors Affecting It in Patients With Type 2 Diabetes Mellitus

**DOI:** 10.7759/cureus.27294

**Published:** 2022-07-26

**Authors:** Tayyab Mumtaz Khan, Fatima Kausar Nawaz, Muhammad Sikandar Karim, Zubair Shafique, Muhammad Saad Anwar, Omer Usman

**Affiliations:** 1 Surgery, Rawalpindi Medical University, Rawalpindi, PAK; 2 Medicine, Shaikh Zayed Hospital, Lahore, PAK; 3 Research, California Institute of Behavioral Neurosciences and Psychology, Fairfield, USA; 4 Internal Medicine, Sahiwal Medical College, Sahiwal, PAK; 5 Neurology, AINeuroCare, Dallas, USA; 6 Internal Medicine, Services Institute of Medical Sciences, Lahore, PAK

**Keywords:** type 2 diabetes mellitus, patients, affecting, factors, microalbuminuria, incidence

## Abstract

Background and objectives

Microalbuminuria prevalence is high in patients with type 2 diabetes mellitus (T2DM) all over the world and its prevalence is affected by several factors. In Pakistan, microalbuminuria and factors that play a role in its development in patients with T2DM are under-researched. This study aimed to determine the incidence of microalbuminuria and the factors affecting it in patients with T2DM.

Material and methods

This descriptive cross-sectional study was performed on 129 diagnosed patients with T2DM in the outpatient department of Benazir Bhutto Hospital, Rawalpindi, for approximately six months from August 2021 to January 2022. Patients were recruited in the study through a non-probability consecutive sampling technique and established inclusion and exclusion criteria. Ethical approval was obtained from the relevant hospital ethical review board (ERB). After explaining the study's aims, informed consent was also taken from all patients before the start of data collection. A self-structured and interview-based questionnaire was used for the collection of data. Descriptive statistics and a chi-square test were applied for the data analysis using Statistical Package for the Social Sciences (SPSS) version 25 (Armonk, NY: IBM Corp.).

Results

The incidence of microalbuminuria in the study population was 31.78%. The association between microalbuminuria and age (p = 0.002), gender (p = 0.003), duration of diabetes mellitus (p = 0.001), therapy type (p = 0.03), control of diabetes mellitus, (p = 0.001), and hypertension (p = 0.002) was statistically significant. Higher age group, male gender, longer duration of diabetes mellitus, oral hypoglycemic agents, poorly controlled diabetes mellitus, and history of hypertension, all were found to raise the incidence of microalbuminuria. Even though being overweight was also found to raise the incidence of microalbuminuria, the association between microalbuminuria and nutritional status was statistically insignificant (p = 0.05).

Conclusion

Microalbuminuria incidence is significantly high in the study population. The factors such as increasing age, male gender, longer duration of the diabetes mellitus, oral hypoglycemic agents, poorly controlled diabetes mellitus, and history of hypertension, all raise the incidence of microalbuminuria in patients with T2DM to a statistically significant extent. Screening of microalbuminuria patients with T2DM should be added to the routine investigations for diabetes mellitus for the early detection of renal and cardiovascular complications.

## Introduction

The prevalence of type 2 diabetes mellitus (T2DM) is high around the globe and it is still increasing. A total of 415 million people had diabetes mellitus in 2015 globally and it has been estimated that this prevalence of diabetes mellitus would reach 642 million by 2040 [1]. Approximately, one in 11 individuals is suffering from diabetes mellitus in the world. In Pakistan, 7.5 million had diabetes mellitus [2].

T2DM is associated with several microvascular and macrovascular complications. Macrovascular complications included myocardial infarction, stroke, and peripheral arterial disease. Microvascular complications lead to retinopathy, neuropathy, and nephropathy. The most common complications of T2DM are related to the cardiovascular system [3]. Modification in diet and lifestyle along with regular medication or insulin injection for hypoglycemic control could prevent these complications.

Abnormal urinary albumin levels occur in 30-40% of patients with T2DM. Screening for microalbuminuria has been recommended by the American Diabetic Association (ADA) for the early detection of renal involvement in T2DM patients [4]. Some studies have also shown that microalbuminuria is an important predictor of mortality and morbidity in T2DM patients from cardiovascular system-related complications [5]. Therefore, prompt management is required for the patients whose samples show microalbuminuria.

Microalbuminuria prevalence is increasing among patients with T2DM and many factors play role in the development of microalbuminuria in patients with T2DM such as age, gender, duration of diabetes mellitus, therapy type, glycemic control, body lipids, smoking, hypertension, and body weight [5-7].

As microalbuminuria in patients with T2DM is understudied in Pakistan and has a high economic impact, we need to take proactive measures to achieve attainable preventive measures. Our study aimed to assess the incidence of microalbuminuria and its association with possible risk factors in patients with T2DM.

## Materials and methods

Study design

This descriptive cross-sectional study was conducted on 129 patients diagnosed with type 2 diabetes mellitus in the outpatient department (OPD) in Benazir Bhutto Hospital (BBH), Rawalpindi, for approximately six months from August 2021 to January 2022.

Inclusion and exclusion criteria

Non-probability convenient sampling technique and developed inclusion and exclusion criteria were applied for the enrollment of the patients. Only those patients who had aged more than 30 years, were diagnosed with T2DM, on oral hypoglycemic agents or insulin, and showed will to participate were included in the study. Those patients who had aged less than 30 years, had no knowledge about the type of diabetes mellitus, diagnosed type 1 diabetes mellitus, active infection, diagnosed congestive cardiac failure, liver disease, high creatinine levels (above 1.3 mg/dL for males and 1.1 mg/dL for females), macroalbuminuria (urine spot for albumin to creatinine ratio above 30 mg/mmol up to 300 mg/mmol), or nephrotic syndrome, current pregnancy, and showed hesitation in participation were excluded from the study.

Data collection

An interview-based and self-structured questionnaire was used to obtain the required data (table in the Appendices). It had two portions. The first portion was about participants' demographic details, including age group (40 and below 40 years or above 40 years) and gender (male or female). The second portion was about clinical data such as duration since diabetes mellitus diagnosis (shorter = less than 10 years, longer = 10 or above years), therapy type (oral hypoglycemic agents, insulin, or both), diabetes mellitus control based on HbA1c and its also known as glycated hemoglobin (well-controlled = 7% and less than 7% or poorly controlled = above 7%), history of hypertension (yes or no), and nutritional status according to World Health Organization (WHO) classification based on body mass index (BMI) (normal when BMI = 18.50-24.90, underweight when BMI = less than 18.50, overweight when BMI = 25.00-29.90, and obese when BMI = 30.00 or above). Height and weight were measured using a measuring tape and a weight machine respectively. BMI was calculated by dividing weight in kilograms (kg) by the square of the height in meters (m). The unit of BMI was kg/m^2^. Microalbuminuria was defined as having a urine spot for albumin to creatinine ratio (UACR) from 3.5 up to 30 mg/mmol in females and from 2.5 mg/mmol up to 30 mg/mmol in males, whereas normoalbuminuria was defined as having UACR less than 3.5 mg/mmol in females and less than 2.5 mg/mmol in males. We collected morning and midstream urine samples, and a UACR was assessed. It was performed in the laboratory of the same hospital and it was noted on the second portion of the questionnaire. 

Ethics 

Ethical approval was obtained for the study from the ethical review board (ERB) of the Benazir Bhutto Hospital, Rawalpindi, and the ethical approval number is BBH.ERB.283/307. Before the start of data collection, informed consent was also acquired from all recruited patients after explaining the aims of the study to them.

Data analysis

After data collection, data analysis was carried out in Statistical Package for the Social Sciences (SPSS) version 25 (Armonk, NY: IBM Corp.). Descriptive analysis was done to calculate the frequencies and percentages of the nominal variables and the means of numerical variables. Chi-square analysis was applied to assess the association between microalbuminuria and potential risk factors. P-value less than 0.05 was set as a significant value.

## Results

Of 129 patients, 62% (n=80) were male and 38% (n=49) were female. Means of age, duration of diabetes, HbA1c, and BMI were 56.43 with standard deviation (SD) of ± 12.20 years, 8.90 with SD of ± 7.23 years, 9.03 with SD of ± 3.01%, and 27.65 with SD of ± 7.73 kg/m^2^ respectively. The overall incidence of microalbuminuria in the study population was 31.78%.

Table 1 describes the association between microalbuminuria and age, gender, duration of diabetes mellitus since diagnosis, therapy type, diabetes mellitus control, and history of hypertension were statistically significant, while the association between microalbuminuria and nutritional status was statistically insignificant. The microalbuminuria was more prevalent among patients who had higher age, male gender, longer duration of diabetes mellitus, oral hypoglycemic agents, poorly controlled diabetes mellitus, history of hypertension, and overweight, in contrast to patients who had lower age, female gender, shorter duration of diabetes, on insulin, well-controlled diabetes mellitus, no history of hypertension, and normal weight.

**Table 1:** Demographic and clinical characteristics of study population along with cross-tabulation and chi-square analysis of study variables

Variables			Cross tabulation and chi-square test		
Total count = 129		n (%)	Albuminuria status		p-Value
			Microalbuminuria, 41 (31.78%)	Normoalbuminuria, 88 (68.22%)	
Age group ( years)	40 and below 40	n = 53 (41.10%)	10 (24.39%)	43 (48.86%)	0.002
	Above 40	n = 76 (58.90%)	31 (75.61%)	45 (51.14%)	
Gender	Male	n = 80 (62.0%)	28 (68.30%)	52 (59.10%)	0.003
	Female	n = 49 (38.0%)	13 (31.70%)	36 (40.90%)	
Duration of diabetes mellitus	Shorter	n = 82 (63.57%)	8 (19.50%)	74 (84.10%)	0.001
	Longer	n = 47 (36.43%)	33 (80.50%)	14 (15.90%)	
Therapy type	Oral hypoglycemic agent	n = 91 (70.55%)	26 (63.41%)	65 (73.86%)	0.03
	Insulin	n = 12 (9.30%)	4 (9.75%)	8 (9.10%)	
	Both	n = 26 (20.15%)	11 (26.84%)	15 (17.04%)	
Diabetes mellitus control	Well-controlled	n = 72 (55.81%)	14 (34.15%)	58 (65.91%)	0.001
	Poorly controlled	n = 57 (44.19%)	27 (65.85%)	30 (34.09%)	
History of hypertension	Yes	n = 61 (47.28%)	29 (70.73%)	32 (36.36%)	0.002
	No	n = 68 (52.72%)	12 (29.27%)	56 (63.64%)	
Nutritional status	Normal	n = 51 (39.54%)	15 (36.60%)	36 (40.90%)	0.05
	Underweight	n = 5 (3.87%)	1 (2.44%)	4 (4.54%)	
	Overweight	n = 60 (46.51%)	21 (51.21%)	39 (44.34%)	
	Obese	n = 13 (10.08%)	4 (9.75%)	9 (10.22%)v	

## Discussion

This study has provided valuable information about the incidence and risk factors of microalbuminuria which predict morbidity and mortality in patients with type 2 diabetes mellitus (T2DM) due to nephropathy and cardiovascular complications.

The incidence of microalbuminuria in the study population was 31.78%. Almost similar incidence of microalbuminuria 31.56% was noted by another study [5]. A lower incidence of microalbuminuria (16.10%) was reported in Iraq [6]. The lower incidence could be due to regional differences in the risk factors of microalbuminuria.

The association between microalbuminuria and age was significant. Microalbuminuria was more prevalent in patients of higher age. This finding of the current study was backed by another study that was conducted in India [8]. Male patients had a higher incidence of microalbuminuria and gender was associated with microalbuminuria significantly. This finding was also recorded in Turkey [9]. Another study presented conflicting findings about gender differences in microalbuminuria and showed a higher prevalence of microalbuminuria in females [5].

Microalbuminuria and duration were associated significantly and microalbuminuria incidence was higher among the patients with a longer duration of the T2DM. This observation was consistent with the result of another research [4]. Types of therapy such as oral hypoglycemic agents and insulin play a crucial role in controlling T2DM. The current study indicated that patients who were on only oral hypoglycemic agents had more incidence of microalbuminuria, and the association between therapy type and microalbuminuria was significant. This finding was also supported by another study [5]. It means that T2DM control through only oral hypoglycemic agents is difficult, so insulin should be added to the treatment regimen whenever T2DM becomes uncontrolled or when the HbA1c value goes above 7% [4].

HbA1c shows the glycemic control of the patients. More than 7% of HbA1c indicates poorly controlled T2DM, while HbA1c less than 7% represents well-controlled T2DM. In this current study, the incidence of microalbuminuria was high in patients with poorly controlled T2DM and the association between the control of T2DM and microalbuminuria was significant. A similar finding was reported by another study that was conducted in Nepal [10].

The role of hypertension in the development of microalbuminuria in patients with T2DM was also noted in this study and microalbuminuria was significantly higher among the patients with a history of hypertension. Some other studies also noted a high prevalence of microalbuminuria in patients with hypertension [5,6].

An increase in body weight was also found to increase microalbuminuria, however, its association with microalbuminuria was statistically insignificant. Another study that was carried out in Nigeria also reported an insignificant association between body mass index and microalbuminuria [7]. However, another study recorded a significant association between body mass index and microalbuminuria [10]. This difference in BMI impact on microalbuminuria could be due to regional body mass index variation in different parts of the world.

Considering the high incidence of microalbuminuria in enrolled patients with T2DM, we recommend screening for microalbuminuria should be added in routine investigations for T2DM, and strategies should be made and implemented for the effective control of all factors that affect microalbuminuria in patients with T2DM to reduce future diabetic complications especially cardiovascular and renal complications.

Since a cross-section study design is used in this study, the temporal association between microalbuminuria and factors affecting it could not be determined. Therefore, further research is required to find out the causal relationship between microalbuminuria and various factors that affect its prevalence.

## Conclusions

The incidence of microalbuminuria in our study was 31.78%. Microalbuminuria was significantly associated with age, gender, duration of diabetes mellitus, therapy type, control of diabetes mellitus, and history of hypertension, while the association between microalbuminuria and nutritional status was statistically insignificant. Patients who had increasing age, male gender, longer duration of diabetes mellitus, on only oral hypoglycemic agents, had poorly controlled diabetes mellitus, history of hypertension, and overweight were found to have more incidence of microalbuminuria than patients who had relatively lower age, female gender, shorter duration of diabetes mellitus, on insulin, well-controlled diabetes mellitus, no history of hypertension, and normal body weight. Diabetes mellitus prevalence is high in our country and hence its associated financial burden is also high. Therefore, vigorous control of diabetes mellitus and its complications is required to avoid future health and economic problems related to diabetes mellitus. One of the key recommendations of the study is that along with adequate control of diabetes mellitus, screening of microalbuminuria should be added in routine investigations for diabetes mellitus. This would lead to early detection of renal and cardiovascular involvement, and after that treatment of that complications would be possible at an early and curable stage.

## Appendices

**Table 2:** Interview-based self-structured research questionnaire

Question number	Research questions	Options: write/tick the option			
1	What is your age?	…..…… in years		Below 40 or 40 years	Above 40 years
2	What is the gender of the patient?	Male		Female	
3	How long have you been diagnosed with diabetes mellitus?	…..…… in years		Shorter = less than 10 years	Longer = more than 10 years
4	What kind of therapy do you use for diabetes mellitus?	Oral hypoglycemic agents only		Inulin	Both
5	What was the value of recent HbA1c?	……… in percentage (%)		Well-controlled diabetes mellitus=7% or less than 7%	Poorly controlled diabetes mellitus = above 7%
6	Do you have previously diagnosed hypertension/raised blood pressure?	Yes		No	
7	What is the height of the patient?	……. in meters (m)			
8	What is the weight of the patient?	……. in kilograms (kg)			
9	What is the body mass index of the patient?	Weight/height in meter^2^…….in kg/m^2^			
10	What is the nutritional status based on the patient's body mass index?	Normal	Underweight	Overweight	Obese
11	What is the urine albumin to creatinine ratio of the patient?	……. in mg/mmol			
12	What is the albuminuria status of the patient based on the urine albumin to creatinine ratio of the patient?	Normoalbuminuria: male = less than 2.5mg/mmol; female = less than 3.5mg/mmol		Microalbuminuria: male = 2.5-30 mg/mmol; female = 3.5-30 mg/mmol	
